# Integrated ionomic and transcriptomic dissection reveals the core transporter genes responsive to varying cadmium abundances in allotetraploid rapeseed

**DOI:** 10.1186/s12870-021-03136-w

**Published:** 2021-08-13

**Authors:** Ting Zhou, Cai-peng Yue, Tian-yu Zhang, Ying Liu, Jin-yong Huang, Ying-peng Hua

**Affiliations:** School of Agricultural Sciences, Zhengzhou University, Zhengzhou, 450001 China

**Keywords:** *B. napus*, Core transporters, Ionome, Transcriptome, Varying cadmium abundances

## Abstract

**Background:**

Oilseed rape (*B. napus* L.) has great potential for phytoremediation of cadmium (Cd)-polluted soils due to its large plant biomass production and strong metal accumulation. Soil properties and the presence of other soluble compounds or ions, cause a heterogeneous distribution of Cd.

**Results:**

The aim of our study was to reveal the differential responses of *B. napus* to different Cd abundances. Herein, we found that high Cd (50 μM) severely inhibited the growth of *B. napus*, which was not repressed by low Cd (0.50 μM) under hydroponic culture system. ICP-MS assays showed that the Cd^2+^ concentrations in both shoots and roots under 50 μM Cd were over 10 times higher than those under 0.50 μM Cd. Under low Cd, the concentrations of only shoot Ca^2+^/Mn^2+^ and root Mn^2+^ were obviously changed (both reduced); under high Cd, the concentrations of most cations assayed were significantly altered in both shoots and roots except root Ca^2+^ and Mg^2+^. High-throughput transcriptomic profiling revealed a total of 18,021 and 1408 differentially expressed genes under high Cd and low Cd conditions, respectively. The biological categories related to the biosynthesis of plant cell wall components and response to external stimulus were over-accumulated under low Cd, whereas the terms involving photosynthesis, nitrogen transport and response, and cellular metal ion homeostasis were highly enriched under high Cd. Differential expression of the transporters responsible for Cd uptake (*NRAMPs*), transport (*IRTs* and *ZIPs*), sequestration (*HMAs*, *ABCs*, and *CAXs*), and detoxification (*MTPs*, *PCR*, *MTs*, and *PCSs*), and some other essential nutrient transporters were investigated, and gene co-expression network analysis revealed the core members of these Cd transporters. Some Cd transporter genes, especially *NRAMPs* and *IRTs*, showed opposite responsive patterns between high Cd and low Cd conditions.

**Conclusions:**

Our findings would enrich our understanding of the interaction between essential nutrients and Cd, and might also provide suitable gene resources and important implications for the genetic improvement of plant Cd accumulation and resistance through molecular engineering of these core genes under varying Cd abundances in soils.

**Supplementary Information:**

The online version contains supplementary material available at 10.1186/s12870-021-03136-w.

## Background

Cadmium (Cd) is a non-essential heavy metal with high biotoxicity for many living organism [1]. Cd has been identified to occur in large quantities of arable land worldwide, and its hyperaccumulation in the edible parts of agricultural crops is causing serious health threat to human beings and animals [2]. Geologically weathering of rocks is a major natural source of Cd contaminants, while the primary anthropogenic sources of Cd, including agrochemicals, manufacturing, vehicular emission, irrigation wastewater, smelting, and mining also resulted in severe Cd pollution [3].

Accumulation of Cd in plants is regulated by several processes, including root adsorption, cell wall retention, xylem loading, vacuolar sequestration, and efflux [4]. When present in ionic form, Cd transport from root to other tissues is mainly mediated by three types of transporters, such as low-affinity calcium (Ca) transporters (LCTs), ZIP [(zinc transporter proteins (ZRT)- and iron-regulated transporter (IRT)-like protein)] transporters, and natural resistance-associated macrophage proteins (NRAMPs) [4]. OsLCT1 localized at the plasma membrane shows Cd efflux activity in yeast, and functions at the nodes in Cd transport into grains [5]. OsNRAMP5, a major influx transporter for Cd, is localized at the distal side of the root epidermis cells [6, 7]. SnYSL3, a member of the Yellow Stripe-Like (YSL) transporters, encodes a plasma-localized transporter delivering Cd-nicotianamine complexes in *Solanum nigrum* [8]. Some Fe^2+^ transporters, such as OsIRT1, OsIRT2, and OsNRAMP1, are potentially involved in Cd uptake [9, 10], whereas they contribute to a small part of Cd uptake.

After entering the roots, a part of Cd is sequestered into root vacuoles by the tonoplast-localized heavy metal ATPase, OsHMA3 [11]. The OsHMA2 transporter is involved in the root-to-shoot translocation of Cd in rice [12]. The cation exchangers (CAX), AtCAX2, and AtCAX4, also transport Cd into vacuoles [13]. In addition to binding to nicotianamine, Cd can also bind with phytochelatins (PCs) that are cysteine-rich polypeptides, which are enzymatically synthesized by the γ-glutamyl-cysteine dipeptide transpeptidase (PC synthase, PCS) [14]. The Cd-PCs complexes formed in the cytosol are transferred to vacuoles by the tonoplast-localized ATP-binding cassette (ABC) transporters, including ABCC1, ABCC2, and ABCC3 [15, 16]. OsABCG36 is reported to be essential for Cd tolerance by exporting Cd or Cd conjugates from rice root cells [17]. Metallothioneins (MTs), also belonging to cysteine-rich protein family members, help plants to store high concentrations of Cd by binding with Cd [4]. Cystein-rich membrane proteins, named as plant Cd resistance (PCR), reduce metal content in plants through increasing heavy metal efflux [18]. The metal tolerance proteins (MTPs), also designated as cation diffusion facilitators (CDFs), are necessary for Cd sequestration or efflux in diverse plants [19]. In addition, exogenous bioactive substances, such as melatonin and glutathione, usually have an obvious effect on Cd accumulation and Cd toxicity resistance [20, 21].

Phytoremediation has been considered as an environmentally friendly and cost-effective approach for removing toxic metals, including Cd, from polluted soils [4]. Previous studies have identified a number of model plants as heavy metal hyperaccumulators, such as *Sedum plumbizincicola*, *Arabidopsis helleri*, and *Noccaea caerulescens* [22, 23]. However, whilst these plant species have strong metal accumulation, they only produce relatively low biomass. This limitation seriously restricts their practical use in the phytoremediation of heavy metal pollutants in the ecosystems.

Soil properties, including the total and available concentration of Cd, pH and organic matter content, cation exchange capacity, clay content, and the presence of other soluble compounds or ions, cause a heterogeneous distribution of Cd, which further lead to distinct responses of plants to varying Cd abundances [24]. High Cd concentrations, but not low Cd, cause severe toxicity symptoms and significantly inhibit plant growth [2]. However, long-term exposure to low Cd concentrations poses a potential threat to human health and plant growth [25].

Allotetraploid rapeseed (*B. napus* L., A_n_A_n_C_n_C_n_, ~ 1130 Mb, 2n = 4x = 38) originated from spontaneous interspecific hybridization between the diploid progenitors *B. rapa* (A_r_A_r_, ~ 485 Mb, 2n = 2x = 20) and *B. oleracea* (C_o_C_o_, ~ 630 Mb, 2n = 2x = 18) about 7500 years ago, followed by chromosome doubling, a process known as allopolyploidy [26]. In addition to being a major vegetable oil source worldwide, oilseed rape shows great potential for phytoremediation by virtue of its large biomass production and strong metal accumulation [27, 28]. Previous studies, focusing on the responses of *B. napus* and other plant species under a certain Cd concentration [29, 30], lack systematic comparative analysis of the molecular responses of plants to both high Cd and low Cd abundances.

Taken together, in this study, we were aimed to investigate the differential ionomic and genome-wide transcriptional responses of allotetraploid rapeseed (A_n_A_n_C_n_C_n_) to varying Cd abundances, and further identify the core Cd transporter gene members responsive to high Cd and low Cd based on the gene co-expression network analysis. The differential expression of Cd transporter genes under high Cd and low Cd conditions might be used to assess the soil Cd abundances. Moreover, our findings would provide suitable gene resources and important implications for the genetic improvement of plant Cd accumulation and resistance through molecular engineering of these genes under varying Cd abundances in soils.

## Results

### Differential ionomic responses of *B. napus* to high cd and low cd abundances

**Fig. 1:**
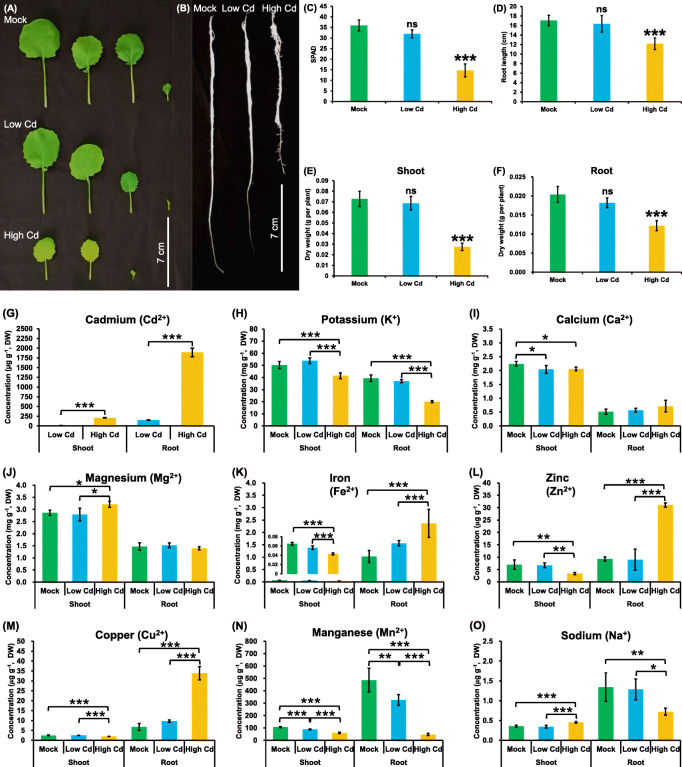
Comparative analysis of growth performance and ion concentrations in rapeseed plants under varying Cd abundances. (**A**-**B**) Shoot (**A**) and root (**B**) growth performance, (**C**) leaf SPAD values, (**D**) root length, and (**E**-**F**) shoot (**E**) and root (**F**) dry weight of rapeseed plants under high Cd and low Cd conditions. (**G**-**P**) The concentrations of Cd^2+^ (**G**), K^+^ (**H**), Ca^2+^ (**I**), Mg^2+^ (**J**), Fe^2+^ (**K**), Zn^2+^ (**L**), Cu^2+^ (**M**), Mn^2+^ (**N**), and Na^+^ (**O**) in rapeseed plants under high Cd and low Cd conditions. Uniform rapeseed plants after 7-day seed germination were grown for 10 d under Cd-free condition, and then the seedlings were transferred to the solution containing Cd-free (0 μM), low Cd (0.50 μM), and high Cd (50 μM CdCl_2_) for 5 days until sampling. Mock means the Cd-free condition; ns, not significant. *, *P* < 0.05; **, *P* < 0.01; ***, *P* < 0.001

In order to assess the responses of *B. napus* to high Cd and low Cd conditions, the plants were grown under a hydroponic culture system. Under low Cd, the rapeseed plants did not show obvious growth defects in both shoots and roots. However, under high Cd, remarkable leaf chlorosis and root inhibition was observed (Fig. 1A, B), which was indicated by smaller SPAD (soil and plant analyzer development) and root length values (Fig. 1C, D). In addition, high Cd also reduced the biomasses in both shoots and roots, which was not significantly changed under low Cd (Fig. 1E, F). Compared with the control (0.28 ± 0.05), high Cd (0.46 ± 0.11) caused an significant increase in the root/shoot ratio, which was not obviously changed under low Cd (0.25 ± 0.04).

Subsequently, the ICP-MS was used to assay the ionomic profiling of several mineral cations, including Cd^2+^, potassium (K^+^), calcium (Ca^2+^), magnesium (Mg^2+^), iron (Fe^2+^), zinc (Zn^2+^), manganese (Mn^2+^), copper (Cu^2+^), and sodium (Na^+^), between high Cd and low Cd abundances. Under both low Cd and high Cd conditions, much higher Cd^2+^ concentrations were observed in the roots than in the shoots (Fig. 1G). Although the Cd^2+^ concentration (50 μM) in the solution under high Cd were 100 fold of that (0.5 μM) under low Cd, the Cd^2+^ concentrations in both shoots and roots under high Cd were over 10 times higher than those under low Cd (Fig. 1G).

In general, according to the responsive patterns of the other eight cations, we divided them into five groups: (i) K^+^, (ii) Ca^2+^ and Mg^2+^, (iii) Fe^2+^, Zn^2+^, and Cu^2+^, (iv) Mn^2+^, and (v) Na^+^ (Fig. 1H-O). The K^+^ concentrations were not significantly changed in both shoots and roots under low Cd, whereas its concentrations were reduced by 15.41 and 70.06% in the shoots and roots, respectively, under high Cd (Fig. 1H). In terms of both Ca^2+^ and Mg^2+^, we observed their decreased concentrations under low Cd but increased concentrations in the shoots under high Cd, whereas no significant changes were found in the roots (Fig. 1I, J). All the concentrations of Fe^2+^, Zn^2+^, and Cu^2+^ were not significantly changed in both shoots and roots under low Cd, whereas their concentrations were significantly reduced in the shoots but were increased in the roots under high Cd (Fig. 1K-M). In the shoots, the Mn^2+^ concentrations were reduced by 32.22 and 16.99% under high Cd and low Cd conditions, respectively (Fig. 1N). In the roots, the Mn^2+^ concentrations were decreased by 85.47 and 33.01% under high Cd and low Cd conditions, respectively (Fig. 1N). The Na^+^ concentrations were not significantly changed in both shoots and roots under low Cd. However, under high Cd, the Na^+^ concentration was significantly increased in the shoots whereas was decreased by 46.15% in the roots (Fig. 1O).

### Overview of the high-throughput transcriptome sequencing data

To investigate the genome-wide transcriptomic responses to high Cd and low Cd conditions, an Illumina HiSeq 4000 system (read length 150 bp, paired end) was used to perform an analysis of high-throughput transcriptional profiling on *B. napus*. After removal of adaptor sequences and low-quality reads, on average, more than 4.7 × 10^7^ clean reads were obtained for each sample, and the total length of clean reads reached 1.2 × 10^9^ nt with the base-calling accuracy of more than 97% Q_20_ and 93% Q_30_ (Supplementary Table S1). In general, all the GC content of 18 RNA samples of rapeseed plants were about 47% in this study. For each sample, ∼90% of the clean reads was mapped to the *B. napus* reference transcriptome sequence.

**Fig. 2:**
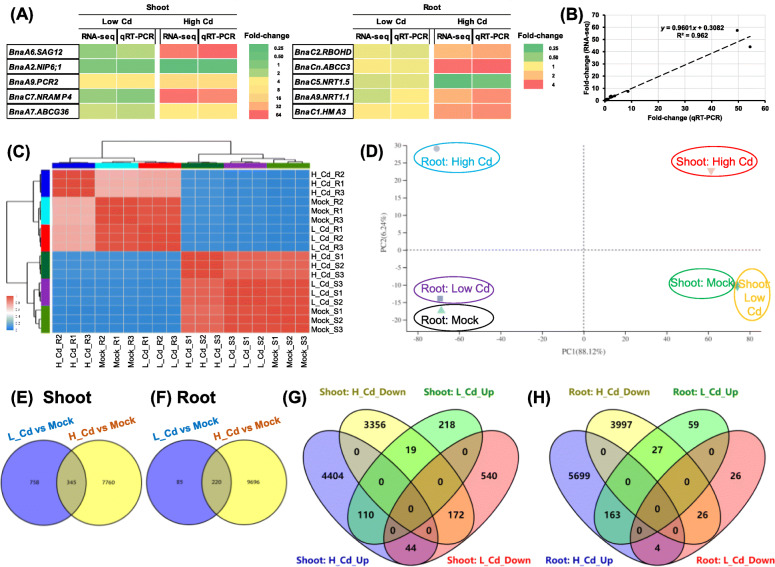
Clustering analysis of differentially expressed genes (DEGs) in the shoots and roots of rapeseed plants under varying Cd abundances. (**A**) Heat maps showing the differential expression fold-changes that were identified by RNA-seq and RT-qPCR assays. (**B**) Consistency analysis of gene expression levels between RNA-seq and RT-qPCR assays. (**C**) Clustering trees among the three biological replicates of each sample. Mock means the Cd-free condition. H_Cd, high Cd; L_Cd, low Cd; S, shoot; R, root. (**D**) Principal component analysis of genome-wide differential gene expression profiling in the shoots and roots of rapeseed plants under high Cd and low Cd conditions. (**E**-**F**) Venn diagram showing DEGs in the shoots (**E**) and roots (**F**) of rapeseed plants under high Cd and low Cd conditions. (**G**, **H**) Venn diagram showing the upregulated and downregulated DEGs in the shoots (**G**) and roots (**H**) of rapeseed plants under high Cd and low Cd conditions

In this study, a total of 10 DEGs, including a *senescence associated gene* (*SAG*) *BnaA6.SAG12*, a boron channel gene *nodulin 26-like intrinsic protein 6;1 (BnaA2.NIP6;1)*, *BnaA9.PCR2*, *BnaC7.NRAMP4*, *BnaA7.ABCG36*, a gene encoding a reactive oxygen species-producing protein *BnaC2.RBOHD*, *BnaCn.ABCC3*, two *nitrate transporter* (*NRT*) genes (*BnaC5.NRT1.5* and *BnaA9.NRTT1.1*), and *BnaC1.HMA3*, were selected to compare their expression correlation between the RT-qPCR assays and transcriptome sequencing. The results showed that the gene expression was highly correlated (*R*^*2*^ > 0.96) between the two assays (Fig. 2A, B). Based on the normalized expression results between two biological replicates, *Pearson* correlation coefficients were calculated, most of which were more than 0.95 (Fig. 2C) between each pair of biological replicates under different Cd treatments. Clustering trees, presenting the distances among biological replicates, showed similar heights among the three biological replicates of each sample. The hierarchical clustering of genome-wide gene expression revealed that a similar expression pattern existed in the three biological replicates of each sample. Taken together, both the analyses of correlation and clustering indicated that the transcriptome sequencing data were of high quality among different biological replicates.

### Genome-scale transcriptomic responses of *B. napus* to high cd and low cd conditions

To investigate the differential molecular responses of *B. napus* to varying Cd abundances, this study was aimed to identify the global differential expression profiling under high Cd and low Cd conditions. In both shoots and roots, different Cd abundances exhibited significantly differential transcriptomic features (Fig. 2D), indicating Cd abundances-dependent transcriptional responses to Cd. Principal component analysis (PCA) showed that the sample distributions on PC1, accounting for 88.12% of total transcriptomic variance, were determined mainly by the rapeseed tissues, including the shoots or roots (Fig. 2D). In other words, the shoots and roots showed significantly distinct responses to high Cd and low Cd. By contrast, the PC2 variance of 6.24% was mainly attributed to varying Cd concentrations. The transcriptional response pattern under high Cd condition was different from that under low Cd, which was similar to the expression pattern under Cd-free condition (Fig. 2D).

In the shoots, a total of 8105 and 1103 genes were identified to be differentially expressed under high Cd and low Cd conditions compared with the Cd-free (mock) condition, respectively (Fig. 2E). In the roots, a total of 9916 and 305 genes were identified to be differentially expressed under high Cd and low Cd conditions compared with the Cd-free (mock) condition, respectively (Fig. 2E). Further, we performed Venn diagram analysis to investigate the responsive patterns of these DEGs under different Cd concentrations. In general, the number of DEGs (471 genes) showing identical expression patterns was identified in both shoots (282 genes) and roots (189 genes) than those of the DEGs (94 genes) showing opposite expression patterns between high Cd and low Cd conditions (Fig. 2F, G).

**Fig. 3:**
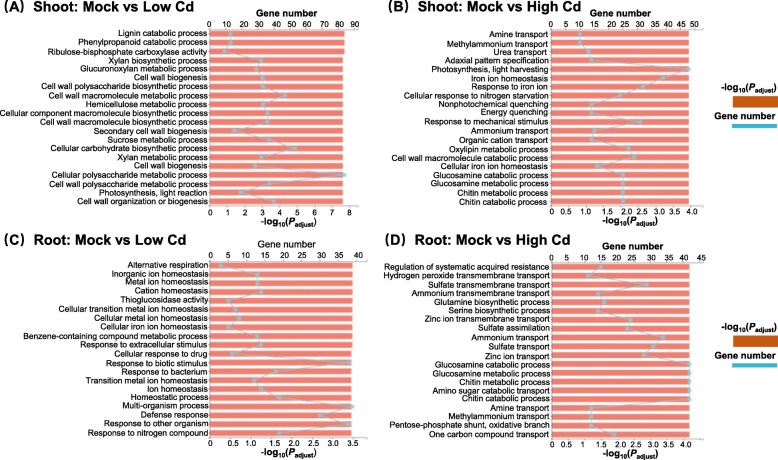
Gene ontology (GO) enrichment analysis of the differentially expressed genes (DEGs) in rapeseed plants under varying Cd abundances. (**A**-**D**) Highly accumulated GO terms in the shoots (**A**, **B**) and roots (**C**, **D**) of rapeseed plants under low Cd (**A**, **C**) and high Cd (**B**, **D**) conditions. Mock means the Cd-free condition

The gene ontology (GO) enrichment analysis of functional significance allowed us to distinguish major biological functions of the DEGs under varying Cd abundances. In the shoots of rapeseed plants under low Cd, we found that the highly enriched GO terms were mainly related to the biosynthesis of plant cell wall components, including lignin, hemicellulose, and xylan (Fig. 3A). However, in the shoots of rapeseed plants under high Cd, the GO terms involving photosynthesis, nitrogen (including amine, urea, and ammonium) transport and response, and response to Fe^2+^ homeostasis were over-accumulated (Fig. 3B). Subsequently, we further investigated the differential GO categories in the roots between high Cd and low Cd conditions. In the roots of rapeseed plants under low Cd, three terms about cellular metal ion homeostasis, response to external stimulus, and defense response were highly accumulated (Fig. 3C). By contrast, the transport and metabolism of Zn^2+^, sulfate, and nitrogen (including ammonium and glutamate) were the most enriched GO groups in the roots of rapeseed plants under high Cd (Fig. 3D).

**Fig. 4:**
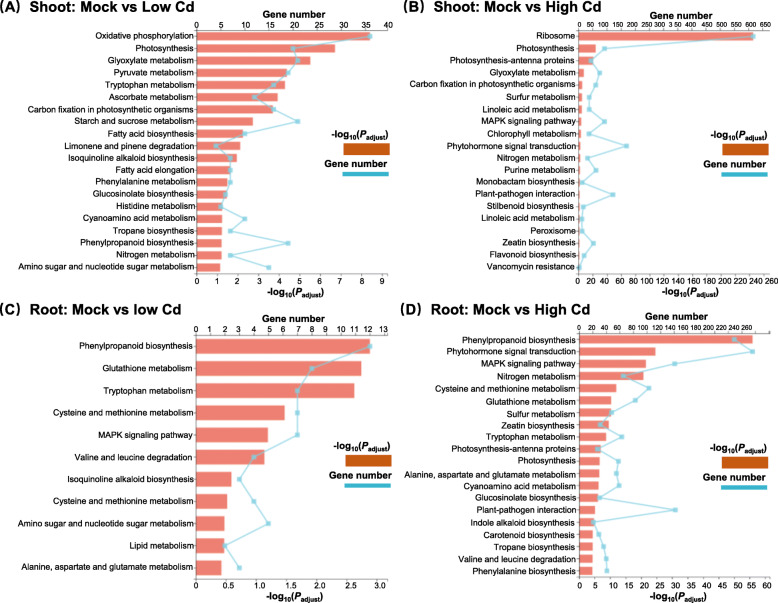
KEGG pathway enrichment analysis of the differentially expressed genes (DEGs) in rapeseed plants under varying Cd abundances. (**A**-**D**) Highly accumulated KEGG pathways in the shoots (**A**, **B**) and roots (**C**, **D**) of rapeseed plants under low Cd (**A**, **C**) and high Cd (**B**, **D**) conditions. Mock means the Cd-free condition

To identify the biological pathways that were active in *B. napus* during exposure to high Cd and low Cd conditions, we characterized the pathways in which the DEGs were involved in the KEGG database. Consistently, photosynthesis in the shoots and phenylpropanoid biosynthesis in the roots were the most active pathways under both high Cd and low Cd conditions (Fig. 4). Compared with the KEGG pathways identified under low Cd, more pathways involving sulfur and nitrogen metabolism, phytohormone biosynthesis and signal transduction were characterized in both shoots and roots under high Cd condition (Fig. 4).

### Genome-wide identification and transcriptional characterization of cd uptake transporters under high cd and low cd conditions

**Fig. 5:**
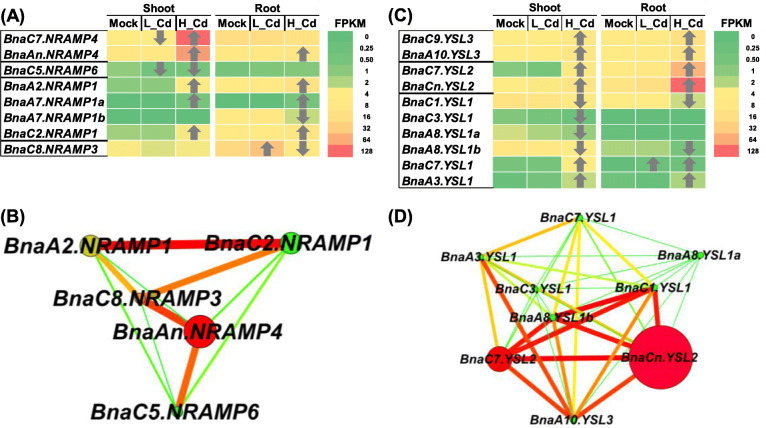
Differential expression and co-expression network analysis of *NRAMPs* and *YSLs* under varying Cd abundances. (**A**-**D**) Differential expression profiling (**A**, **C**) and co-expression network analysis (**B**, **D**) of *NRAMPs* (**A**, **B**) and *YSLs* (**C**, **D**) in the shoots and roots of rapeseed plants under high Cd and low Cd conditions. In the heat maps, the upward and downward arrows indicate the upregulation and downregulation of differentially expressed genes. In the gene co-expression networks, cycle nodes represent genes, and the size of the nodes represents the power of the interrelation among the nodes by degree value. Edges between two nodes represent interactions between genes. Mock means the Cd-free condition

In the allotetraploid rapeseed genome (A_n_A_n_C_n_C_n_), a total of 22 *NRAMP* transporter genes, including *NRAMP1* to *NRAMP6*, were functionally annotated and identified in the transcriptome sequencing data. In this study, only eight members of the *NRAMP* families were identified to be differentially expressed under high Cd and low Cd (Fig. 5A). Among the *NRAMP* DEGs, it is noteworthy that both *BnaC7.NRAMP4* (BnaC07g15960D) and *BnaC8.NRAMP3* (BnaC08g34570D) were differentially responsive to high Cd and low Cd conditions. The expression of *BnaC5.NRAMP6* (BnaC05g12190D) was repressed under both high Cd and low Cd conditions. In addition, the other *NRAMP* DEGs were upregulated in both shoots and roots under high Cd. According to the gene co-expression network analysis result, *BnaAn.NRAMP4* (BnaAnng14550D) was proposed to be the central *NRAMP* family member (Fig. 5B).

Among the genome-wide 27 *YSLs* that were identified in *B. napus*, *BnaC7.YSL1* (BnaC07g38730D), the only one *YSL* homolog responsive to low Cd, was upregulated in the shoots (Fig. 5C). Among the DEGs responsive to high Cd, four *BnaYSL1s* (*BnaC1.YSL1/*BnaC01g15940D,*BnaC3.YSL1/*BnaC03g62050D,*BnaA8.YSL1a/*BnaA08g15130D, and *BnaA8.YSL1b/*BnaA08g10710D) were downregulated only in the shoots, and other *BnaYSL* DEGs were induced by high Cd in both shoots and roots. The two homologs of *YSL2s* showed the highest expression abundances and largest fold-changes under high Cd, especially in the roots. The gene co-expression network analysis showed that *BnaCn.YSL2* (BnaCnng70180D) might be the core *YSL* family gene (Fig. 5D).

**Fig. 6:**
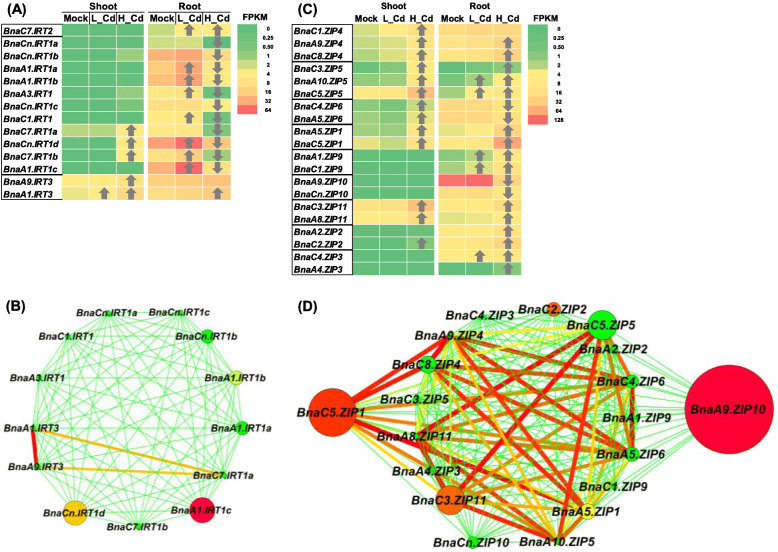
Differential expression and co-expression network analysis of *IRTs* and *ZIPs* under varying Cd abundances. (**A**-**D**) Differential expression profiling (**A**, **C**) and co-expression network analysis (**B**, **D**) of *IRTs* (**A**, **B**) and *ZIPs* (**C**, **D**) in the shoots and roots of rapeseed plants under high Cd and low Cd conditions. In the heat maps, the upward and downward arrows indicate the upregulation and downregulation of differentially expressed genes. In the gene co-expression networks, cycle nodes represent genes, and the size of the nodes represents the power of the interrelation among the nodes by degree value. Edges between two nodes represent interactions between genes. Mock means the Cd-free condition

In this study, we identified a total of 14 *IRT* DEGs, which included 11 *IRT1s*, one *IRT2*, and two *IRT3s*, under high Cd and low Cd conditions (Fig. 6A). All the nine *IRT* DEGs that were identified under low Cd were upregulated, however, most of the 14 *IRT* DEGs that were identified under high Cd were downregulated, particularly in the roots (Fig. 4A). The gene co-expression network analysis showed that *BnaA1.ITRT1c* (BnaA01g35020D) might be the core *IRT* family gene (Fig. 6B).

In *B. napus*, we retrieved a total of 53 *ZIP* family genes. Based on the transcriptome sequencing data, we identified 20 *BnaZIP* DEGs, five members of which were only responsive to low Cd. Among all the DEGs, a major proportion (80%) was upregulated in the shoots or roots under high Cd except *BnaC4.ZIP6* (BnaC04g14740D), *BnaA5.ZIP6* (BnaA05g12290D),*BnaA9.ZIP10*(BnaA09g25300D), and *BnaCn.ZIP10* (BnaC04g14740D) that were repressed by high Cd only in the roots (Fig. 6C). Among the upregulated *BnaZIP* DEGs, *BnaC5.ZIP1* (BnaC05g40440D) and *BnaA9.ZIP10* (BnaA09g25300D) showed the highest expression abundances and largest fold-changes under high Cd, and they were identified to be core members of *BnaZIPs* (Fig. 6D).

### Genome-wide identification and transcriptional characterization of vacuolar cd transporters under high cd and low cd conditions

**Fig. 7:**
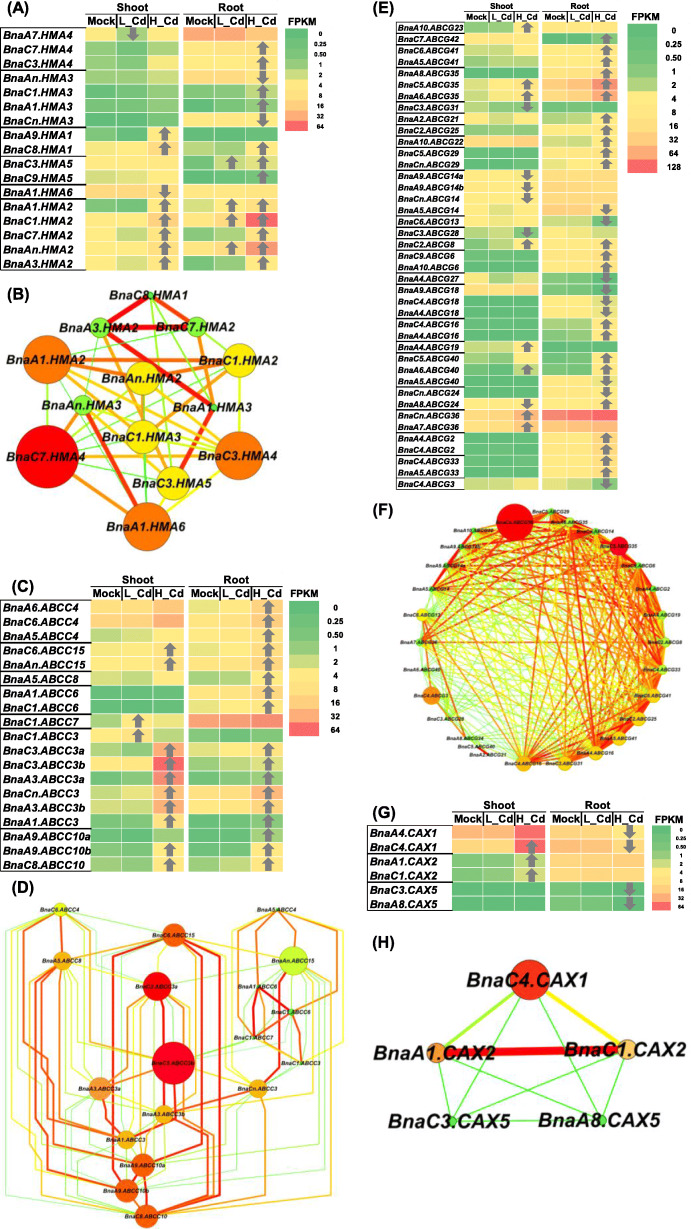
Differential expression and co-expression network analysis of *HMAs*, *ABCCs*, and *CAXs* under varying Cd abundances. (**A**-**H**) Differential expression profiling (**A**, **C**, **E**, **G**) and co-expression network analysis (**B**, **D**, **F**, **H**) of *HMAs* (**A**, **B**), *ABCCs* (**C**, **D**), ABCGs (**E**, **F**) and *CAXs* (**G**, **H**) in the shoots and roots of rapeseed plants under high Cd and low Cd conditions. In the heat maps, the upward and downward arrows indicate the upregulation and downregulation of differentially expressed genes. In the gene co-expression networks, cycle nodes represent genes, and the size of the nodes represents the power of the interrelation among the nodes by degree value. Edges between two nodes represent interactions between genes. Mock means the Cd-free condition

Among the genome-wide 32 *HMAs* in *B. napus*, a total of 17 *HMA* DEGs were identified. Among these DEGs, in general, the *HMA2* subfamily members showed much higher expression abundances than other subfamily members (Fig. 7A). Only four DEGs (*BnaA7.HMA4/*BnaA07g36130D,*BnaAn.HMA3/*BnaAnng10870D,*BnaCn.HMA3/*BnaCnng78610D, and *BnaA1.HMA6/*BnaA01g03390D) were downregulated in the shoots or roots under high Cd or low Cd, whereas the expression of other *HMA* DEGs was induced in both shoots and roots under high Cd (Fig. 7A). Gene co-expression network analysis showed that *BnaA1.HMA2* (BnaA01g06430D) and *BnaC7.HMA4* (BnaC07g02470D) might play a core role in *HMA*-mediated Cd transport (Fig. 7B).

In *B. napus*, a total of 314 ABC transporter genes were identified, which included 47 *ABCC* members [31]. Among these ABCCs, there were two and 17 DEGs identified under low Cd and high Cd conditions, respectively (Fig. 7C). In general, the ABCC3 DEGs showed higher expression levels than other members. Under low Cd, two DEGs, *BnaC1.ABCC7* (BnaC01g37990D) and *BnaC1.ABCC3* (BnaC01g37970D), were upregulated only in the shoots, whereas they showed no significant changes under high Cd. Under high Cd, all the 17 DEGs were upregulated, whereas they were not differentially expressed under low Cd. Through gene co-expression network analysis, *BnaC3.ABCC3b* (BnaC03g73620D) was identified to be the core ABCC-mediated gene regulating vacuolar Cd sequestration (Fig. 7D). Further, we investigated the differential expression of *ABCGs* under high Cd and low Cd conditions. The result showed that all the 41 DEGs that were identified were only responsive to high Cd (Fig. 7E). Among the *ABCG* DEGs, the *ABCG35* and *ABCG36* subgroups showed the highest expression levels. A larger proportion of the DEGs were upregulated in both shoots (57%) and roots (72%) under high Cd. Through gene co-expression network analysis, *BnaCn.ABCG36* (BnaCnng64010D) and *BnaC5.ABCG35* (BnaC05g11500D) were identified to be the core *ABCC*-mediated genes regulating Cd efflux in the shoots and roots, respectively (Fig. 7F).

According to the rapeseed genome information, we retrieved a total of 17 *CAX* family genes. All the genome-wide differentially expressed *BnaCAXs* were identified under high Cd, whereas low Cd did not cause the differential expression of *BnaCAXs* (Fig. 7G). In general, the CAX1 subgroup members had much higher expression abundances than other subgroup. In the shoots, all the three identified *CAX* DEGs, including *BnaC4.CAX1* (BnaC04g45720D), *BnaA1.CAX2* (BnaA01g29870D), and *BnaA1.CAX2* (BnaC01g37750D), were upregulated. In the roots, all the four identified *CAX* DEGs, including *BnaA4.CAX1* (BnaA04g21850D), *BnaC4.CAX1* (BnaC04g45720D), *BnaC3.CAX5* (BnaC03g70750D), and *BnaA8.CAX5* (BnaA08g00350D), were downregulated. Among the six *CAX* DEGs, only *BnaC4.CAX1* was differentially expressed in both shoots and roots, whereas it showed opposite expression patterns between shoots (upregulated) and roots (downregulated) (Fig. 7H).

### Genome-wide identification and transcriptional characterization of other cd transporters under high cd and low cd conditions

**Fig. 8:**
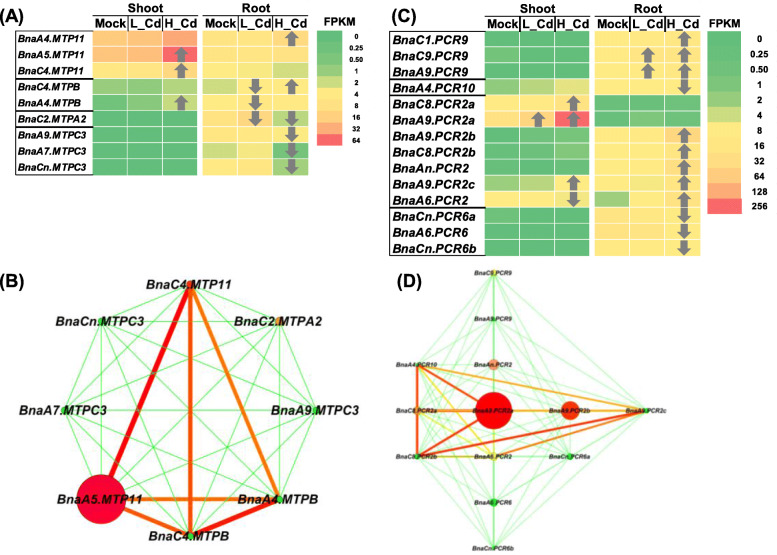
Differential expression and co-expression network analysis of *MTPs* and *PCRs* under varying Cd abundances. (**A**-**D**) Differential expression profiling (**A**, **C**) and co-expression network analysis (**B**, **D**) of *MTPs* (**A**, **B**) and *PCRs* (**C**, **D**) in the shoots and roots of rapeseed plants under high Cd and low Cd conditions. In the heat maps, the upward and downward arrows indicate the upregulation and downregulation of differentially expressed genes. In the gene co-expression networks, cycle nodes represent genes, and the size of the nodes represents the power of the interrelation among the nodes by degree value. Edges between two nodes represent interactions between genes. Mock means the Cd-free condition

In allotetraploid *B. napus*, a total of 33 *MTPs* were annotated [32], and there were three and nine members differentially expressed under low Cd and high Cd conditions, respectively. Under low Cd, three *MTP* DEGs (*BnaC4.MTPB/*BnaC04g40510D, *BnaA4.MTPB/*BnaA04g28640D, and *BnaC2.MTPA2/*BnaC02g06740D) were identified, and all of them were downregulated. Under high Cd, a total of *MTP* DEGs were characterized, four and five of which were upregulated and downregulated, respectively (Fig. 8A). It was noteworthy that two *BnaMTPB* DEGs showed significantly differential responsive patterns under high Cd and low Cd conditions: both of them were downregulated under low Cd whereas they were upregulated under high Cd (Fig. 8A). Gene co-expression network analysis showed that *BnaA5.MTP11* was the core family gene (Fig. 8B), which presented the highest expression level and largest fold-change (Fig. 8A).

In Arabidopsis, the *PCR* family contained 12 subgroups, namely *PCR1*-*PCR12* (https://www.arabidopsis.org/index.jsp). In this study, three *PCR* DEGs were identified under low Cd, and all of them were upregulated. By contrast, a total of 14 *PCR* DEGs were characterized under high Cd, and 10 (71.42%) of them were upregulated except the downregulated *BnaPCR6* homologs and *BnaA4.PCR10* (BnaA04g23620D) (Fig. 8C). Based on the gene co-expression network analysis, *BnaA9.PCR2a* (BnaA09g45360D) was proposed to be the central *PCR* member (Fig. 8D).

### Genome-wide identification and transcriptional characterization of cd chelators under high cd and low cd conditions

**Fig. 9:**
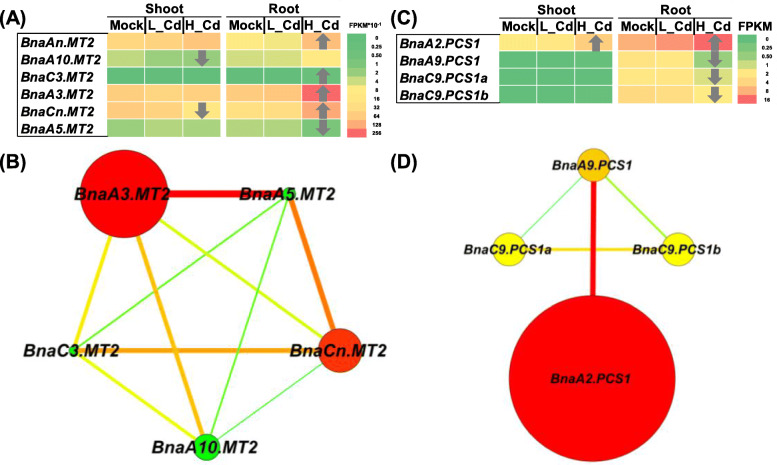
Differential expression and co-expression network analysis of *MTs* and *PCSs* under varying Cd abundances. (**A**-**D**) Differential expression profiling (**A**, **C**) and co-expression network analysis (**B**, **D**) of *MTs* (**A**, **B**) and *PCSs* (**C**, **D**) in the shoots and roots of rapeseed plants under high Cd and low Cd conditions. In the heat maps, the upward and downward arrows indicate the upregulation and downregulation of differentially expressed genes. In the gene co-expression networks, cycle nodes represent genes, and the size of the nodes represents the power of the interrelation among the nodes by degree value. Edges between two nodes represent interactions between genes. Mock means the Cd-free condition

Plant *MTs* are classified into four types according to the arrangement of their cystein residues [33], including the *MT1*, *MT2*, *MT3*, and *MT4* subfamilies [34]. However, we identified the differential expression of *MTs* only under high Cd (Fig. 9A). Among the six *BnaMT2* DEGs, three *BnaMT2s* were downregulated and four *BnaMT2s* were upregulated in the shoots or roots. Gene co-expression network analysis revealed that *BnaA3.MT2*, showing the highest expression level and largest fold-change, was identified to be the core member of MTs (Fig. 9B).

In the genome-wide *BnaPCS* family genes, we only identified four DEGs, all of which were responsive to high Cd (Fig. 9C). However, three of them, including *BnaA9.PCS1*, *BnaC9.PCS1a*, and *BnaC9.PCS1b*, were downregulated, and only *BnaA2.PCS1* was upregulated in both shoots and roots under high Cd. Both gene expression pattern and co-expression network analysis revealed that *BnaA2.PCS1* was the core gene that potentially functioned in the Cd detoxification process (Fig. 9D).

### Genome-wide transcriptional characterization of other element transporters under high cd and low cd conditions

**Fig. 10:**
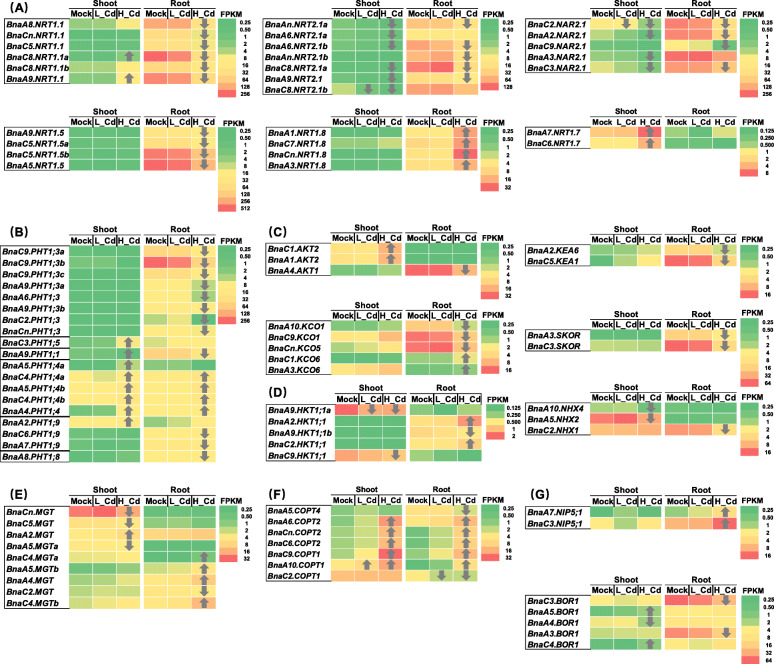
Differential expression of some essential element transporter genes under varying Cd abundances. (**A**-**F**) Differential expression profiling of transporter genes of nitrate (**A**), phosphate (**B**), potassium (**C**), sodium (**D**), magnesium (**E**), copper (**F**), and boron (**G**) nutrients in the shoots and roots of rapeseed plants under high Cd and low Cd conditions. In the heat maps, the upward and downward arrows indicate the upregulation and downregulation of differentially expressed genes. Mock means the Cd-free condition

In order to identify the effect of exogenous vary Cd abundances on other essential element metabolism, genome-wide transcriptional profiling of the transporter genes of nitrate, phosphate, potassium, sodium, magnesium, copper, and boron nutrients (Fig. 10). In general, the expression of these above-mentioned transporter genes was not significantly changed under low Cd, whereas high Cd induced extensive alteration of their transcriptional levels (Fig. 10). Under both high Cd and low Cd conditions, the DEGs of dual-affinity nitrate transporter genes (*BnaNRT1.1 s/BnaNPF6.3 s*) and two-component high-affinity nitrate transporter genes (*BnaNRT2.1 s* and *BnaNAR2.1 s/BnaNRT3.1 s*) were downregulated (Fig. 10A). Meanwhile, high Cd repressed the expression of xylem-loading nitrate transporter genes (*BnaNRT1.5 s/BnaNPF7.3 s*) whereas induced the expression of xylem-unloading nitrate transporter genes (*BnaNRT1.8 s/BnaNPF7.2 s*) (Fig. 10A). The expression of *NRT1.7/NPF2.13* responsible for source-to sink remobilization of nitrate was increased in the shoots under high Cd (Fig. 10A). Under high Cd, the expression of phosphate transporter genes, including *BnaPHT1;1 s*, Bna*PHT1;3 s*, *BnaPHT1;8 s*, and *BnaPHT1;9 s*, was downregulated in the roots. However, the expression levels of *BnaPHT1;4 s* were significantly increased in both shoots and roots (Fig. 10B). The differential expression of some potassium transporter genes, including the chloroplast-localized K^+^ efflux transporter gene *KEA* (*K*^*+*^ *efflux antiporter*), the vacuolar K^+^ influx transporter gene *KCO* (*two-pore K*^*+*^ *channel*), the plasma membrane-localized K^+^ influx transporter genes *AKT* (*Arabidopsis K*^*+*^ *transporter*) and, and the K^+^ efflux gene *SKOR* (*stelar K*^*+*^ *outward rectifier*), was also observed only under high Cd, and most of the DEGs were downregulated particularly in the roots (Fig. 10C). *HKT1* (*high-affinity K*^*+*^ *transporter 1*) is reported to be involved in the root xylem Na^*+*^ unloading in dicots, and the expression of *BnaHKT1s* was increased mainly in the roots, and the vacuolar Na^+^ influx transporter genes, including *BnaC2.NHX1*, *BnaA5.NHX2*, and *BnaA10.NHX4*, were downregulated (Fig. 10D). In terms of the magnesium transporter (MGT) genes, the *MGT* DEGs were upregulated mainly in the roots and were downregulated mainly in the shoots (Fig. 10E). The general expression of copper transporter (COPT) genes was increased in both shoots and roots under high Cd (Fig. 10F). Eventually, we investigated the differential expression of a root boron influx channel gene *NIP5;1* and a root xylem boron loading transporter gene *BOR1*, and the RNA-seq result showed that *BnaNIP5;1 s* and *BnaBOR1s* were upregulated and downregulated in the roots, respectively (Fig. 10G).

## Discussion

Contaminated soils in the environment usually have a heterogeneous distribution of heavy metals [35], which leads to high and low metal abundances at different soil sites. Previous studies, mainly focusing on the responses of plants to high Cd, lack systematic dissection into the differential responses of plants to high Cd and low Cd abundances. In the study, *B. napus*, a promising metal-hyperaccumulating crop species, was hydroponically grown under high Cd and low Cd conditions. Subsequently, the ionomic homeostasis and transcriptomic profiling were investigated to achieve an understanding of differential physiological and molecular responses to varying Cd abundances and identify the core Cd transporter genes among multi-copy gene families in allotetraploid rapeseed. The findings obtained in this study can be used as a guidance for the genetic improvement of plant Cd resistance and accumulation under high Cd and low Cd conditions.

### Differential ionomic responses of *B. napus* to high cd and low cd imply complex interaction between nutrient elements and cd

Under both low Cd and high Cd conditions, much higher Cd concentrations were observed in the roots than in the shoots (Fig. 1G), and it might be a smart defensive reaction against Cd: plants maintain shoot growth and photosynthesis through root Cd retention. The differences in the Cd concentrations within rapeseed plants were much smaller than those in the hydroponic solution between high Cd and low Cd conditions (Fig. 1G). In this study, the Cd^2+^ concentration of 0.50 μM did not cause severe phytotoxicity, which might not restrain the Cd accumulation in rapeseed plants. Therefore, the smaller differences in the tissue Cd concentrations might be attributed to low Cd accumulation of rapeseed plants under high Cd, which could be thought of to be a smart defensive reaction of rapeseed plants against Cd toxicity.

According to the ion-responsive patterns under high Cd and low Cd, the other eight cations were categorized into five groups: (i) K^+^, (ii) Ca^2+^ and Mg^2+^, (iii) Fe^2+^, Zn^2+^, and Cu^2+^, (iv) Mn^2+^, and (v) Na^+^ (Fig. 1H-O). The K^+^ concentrations, hardly affected by low Cd, were significantly decreased in both shoots and roots under high Cd (Fig. 1H), and it indicated that high Cd severely inhibited total K^+^ uptake, which was confirmed by the decreased expression of *BnaAKTs* in the roots (Fig. 10C). Therefore, increasing K^+^ supply is a strategy to improve the tolerance of plants exposed to high Cd toxicity [36], and K^+^-mitigated high Cd toxicity is related to the enhancement of Cd fixation in the root cell wall [37]. In terms of both Ca^2+^ and Mg^2+^ in the shoots, their concentrations were significantly decreased under low Cd whereas were significantly increased under high Cd (Fig. 1I, J). However, the concentrations of root Ca^2+^ and Mg^2+^ showed no significant changes under both high Cd and low Cd (Fig. 1I, J), which indicated that high Cd contributed to the translocation of Ca^2+^ and Mg^2+^ from roots to shoots, whereas this process was repressed by low Cd. CaCl_2_ induces reduction of Cd accumulation, improves cell membrane stability, and increases the antioxidant defense systems, thus alleviating high Cd toxicity [38]. Exogeneous Mg supply not only relieves symptoms of high Cd-induced toxicity by altering the expression of Cd-induced genes, but also inhibits Cd translocation from roots to shoots [39]. The concentrations of Fe^2+^, Zn^2+^, and Cu^2+^ were not significantly affected by low Cd, however, their concentrations were significantly reduced in the shoots but were increased in the roots under high Cd (Fig. 1K-M). This result suggested that high Cd reduced the translocation of Fe^2+^, Zn^2+^, and Cu^2+^ from roots to shoots. Moderate Fe application eliminates Cd-induced decrease in net photosynthetic rate as well as the disorder of antioxidant systems [40]. Foliar Zn application reduces root Cd translocation to shoots, while soil Zn application contributes to the reduction of root Cd concentrations [41]. Under both high Cd and low Cd, only the concentrations of Mn^2+^ were remarkably changed in both shoots and roots (Fig. 1N), which implied close interactions occurred between Cd and Mn. Although the Mn^2+^ uptake was significantly inhibited by both high Cd and low Cd, both of which contributed to the translocation of Mn^2+^ from roots to shoots (Fig. 1N). Similarly, Mn supply significantly decreased Cd bioaccumulation in other plant species, including *Celosia argentea*, *Phytolacca americana*, and *Lupinus albus* [40, 42, 43]. The concentrations of Na^+^, functioning as a beneficial element under low concentrations for plants [44], were not significantly changed in both shoots and roots under low Cd (Fig. 1O). However, its concentration was significantly increased in the shoots but was decreased in the roots under high Cd (Fig. 1O), which suggested that high Cd favored the translocation of Na^+^ from roots to shoots. NaCl addition in the Cd-containing medium caused remarkable reductions in both Cd concentration and accumulation [3].

As a non-essential element, Cd may share a portion of the other metal transporters in plants [40], whereas specific Cd transporters have been not identified so far. The Cd-induced inhibited uptake or translocation of other metal ions may be attributed to the antagonism [45]. The interaction between Cd and other metal ions could be used as guidance for the enhancement of plant Cd resistance and hyperaccumulation-based Cd phytoremediation in agricultural practice.

### Co-expression network assisted analysis of transcriptomic responses to high cd and low cd reveals core cd transporter genes

Previous studies mainly focus on the responses of plants to high Cd toxicity [4], whereas heterogenous Cd conditions, including high phytotoxic Cd abundance and low Cd without obvious phytotoxicity, usually occur in soils [46]. In polyploid *B. napus*, multiple-copy gene families are common; therefore, identification of the core gene(s) is a key prerequisite for the understanding of molecular mechanisms underlying important agronomy traits. Therefore, systematic analysis of plant transcriptional responses to high Cd and low Cd and molecular characterization of Cd transporter genes will give us a comprehensive understanding of plant adaptation to heterogenous Cd conditions. The findings will provide elite gene resources for the genetic improvement of plant Cd resistance and hyperaccumulation-based Cd phytoremediation.

The result of principal component analysis revealed that the shoots and roots of rapeseed plants showed significantly distinct responses to high Cd and low Cd conditions (Fig. 2D). In this study, different Cd abundances exhibited significantly differential transcriptomic features in both shoots and roots of rapeseed plants (Fig. 2D), which indicated the abundances-dependent transcriptional responses of rapeseed to Cd. Moreover, through principal component analysis (Fig. 2D), we proposed that the rapeseed tissues had a more pronounced effect on transcriptional features than the Cd abundances. In this study, more DEGs were identified in both shoots and roots of rapeseed plants under high Cd than those under low Cd (Fig. 2E-G), which suggested that high Cd had a more significant effect on the growth and molecular responses in *B. napus* than low Cd.

Plant roots are the first organ that directly interacts with soil environmental stresses [47]. In the roots of rapeseed plants under low Cd, the GO term involving response to external stimulus and the KEGG pathways involving MAPK signaling transduction were highly accumulated (Figs. 3C, 4C). Therefore, we presumed that low Cd triggered the defense response of rapeseed plants through activating MAPK signaling pathways (Fig. 1) although low Cd did not cause obvious leaf Cd toxicity (Fig. 1A). The overaccumulation of photosynthesis-related KEGG terms under low Cd might be attributed to the fact that low Cd disturbed the homeostasis of Mg^2+^ and Mn^2+^ (Fig. 1J, N), both of which are key to photosynthesis [48]. Sulfur (S), an essential element for plants, participates in the metabolism of methionine, cysteine, glutathione, and phytochelatin [49]. Cell wall functions as an important physical barrier to protect plants from heavy metal contaminants by reducing uptake or preventing entry into the cytoplasm [50]. Meanwhile, cell wall is also a major site for Cd sequestration and accumulation [29]. Both GO and KEGG enrichment analysis showed that the metabolism of sulfide, including cysteine, methionine, and glutathione and phenylpropanoid biosynthesis, was the highly enriched group in the roots of rapeseed plants under high Cd (Figs. 3D, 4D), which highlighted that the pivotal roles of sulfur-mediated chelation and cell wall-mediated retention in Cd hyperaccumulation and Cd resistance. In the shoots of rapeseed plants under low Cd, the highly enriched GO and KEGG terms were mainly related to the biosynthesis of plant cell wall components (Figs. 3A, 4A). It enlightened us that regulation of cell wall metabolism might contribute to Cd hyperaccumulation in cell walls of plants, which is favorable for phytoremediation of Cd pollutants. However, in the shoots of rapeseed plants under high Cd, the GO and KEGG terms involving photosynthesis, nitrogen transport and response, and response to ion homeostasis were over-accumulated (Figs. 3B, 4B), which indicated that high Cd toxicity might mainly alter the expression of genes related to photosynthesis and nutrient metabolism.

The polyploidy events within the *B. napus* genome result in numerous duplicated segments and homoeologous regions, which cause formation of multi-copy gene families. Furthermore, the core Cd transporter genes within the rapeseed genome remain largely unknown, which greatly restricts the understanding of responsive mechanisms underlying strong Cd resistance and high Cd accumulation in rapeseed. In this study, based on gene functional annotation and co-expression networks, some core Cd transporter genes were identified, and these genes were presumed to main regulators responsible for Cd uptake and translocation. Therefore, integrated genomics and transcriptomics analysis might be an efficient pathway that was feasible for the rapid identification of core genes without performing genome-wide association study or map-based gene cloning.

In this study, in terms of the *NRAMPs* mainly responsible for Cd uptake and transport, we found that *BnaNRAMP5s* had very low expression levels even under high Cd, and did not identify the differential expression of *BnaNRAMP5s* under both high Cd and low Cd conditions (Fig. 5A). It indicated that *NRAMP5s* might not be involved in the Cd uptake and transport. Moreover, we found that some Cd transporters, such as *BnaC7.NRAPM4*, *BnaC8.NRAPM3*, and some *BnaIRT1s*, showed opposite expression patterns between high Cd and low Cd conditions (Figs. 5A, 6A).

In addition to impacting other metal homeostasis, we presumed that Cd also had a significant effect on metabolism of non-metal elements based on the transcriptome and RT-qPCR results. For example, the expression of nitrate and boron transporters was significantly changed, particularly under high Cd toxicity (Figs. 2A, 10A, G). In terms of nitrate transporters, NRT1.1/NPF6.3 is a dual-affinity nitrate influx transporter in the roots of plants [51], and NRT1.5/NPF7.3 is mainly responsible for root xylem nitrate loading [52]. In this study, both transcriptome and RT-qPCR results showed that the expression of *BnaA9.NRT1.1* and *BnaC5.NRT1.5* was repressed by high Cd toxicity (Fig. 2A). In Arabidopsis, it has been found that Cd inhibits nitrate uptake and inhibits the expression of *NRT1.1*, and loss of *NRT1.1* function alleviates the phytotoxicity caused by Cd [53]. Increased nitrate is allocated to roots of the *atnrt1.5* mutant, which shows stronger Cd resistance compared with the wide type [54]. Taken together, there may be a close interaction between nitrogen nutrients and Cd toxicity resistance. In addition, boron homeostasis might be also greatly affected by Cd. In this study, the expression of a root boron influx channel NIP5;1 [55], induced by boron deficiency [56], was downregulated by high Cd toxicity (Fig. 10G), which suggested that Cd repressed the boron uptake. However, the expression of both a root xylem boron loading BOR1 [57] and a boron channel responsible for preferential transport of boron to growing shoot tissues, NIP6;1 [58], was significantly decreased under both high Cd and low Cd conditions (Figs. 2A, 10G). It suggested that Cd might inhibit the root xylem boron loading and boron recycling from source leaves to sinks. Previous studies show increased boron supply alleviates Cd toxicity through inhibiting Cd uptake and increasing cell wall-mediated Cd retention [59, 60]. Therefore, application of boron fertilizers could be used to reduce Cd accumulation and enhance Cd resistance.

This finding suggested that different strategies should be adopted through molecular modulation of Cd transporters for the genetic improvement of plant Cd resistance and phytoremediation under different Cd abundances. For example, enhancing the expression of *BnaIRT1s* might be favorable for hyperaccumulation-based Cd phytoremediation under low Cd, whereas reducing the expression of *BnaIRT1s* might contribute to improving plant Cd resistance.

## Conclusions

Taken together, in this study, we presented the differential genome-wide transcriptional responses of allotetraploid rapeseed (A_n_A_n_C_n_C_n_) to varying Cd abundances, which greatly eased the identification of core Cd transporter gene members responsive to high Cd and low Cd based on the gene co-expression network analysis. Our findings will provide suitable gene resources and important implications for the genetic improvement of plant Cd accumulation and resistance through molecular engineering of these genes under varying Cd abundances in soils.

## Materials and methods

### Plant materials and growth condition

Considering the rapeseed cultivar of Zhongshuang 11 (a winter cultivar), having well-known information on genome sequences, is an elite genotype with high oil quality, seed production [61], and strong Cd resistance [30], we used Zhongshuang 11 as the rapeseed lines studied in the following experiments. Due to high homogeneity of culture media and easy management, hydroponic culture was used as the rapeseed-growing way, which is more suitable for this study than soil culture and tissue culture. *B. napus* seedlings, the seeds of which were collected from Prof. Jin-yong Huang (jinyhuang@zzu.edu.cn, Zhengzhou University, Zhengzhou, 450,001, China), were grown in an illuminated growth chamber using the Hoagland nutrient solution. Growth conditions were set as follows: light intensity of 150 μmol m^− 2^ s^− 1^, room temperature of 24 °C daytime/22 °C night, light period of 16-h photoperiod/8-h dark, and relative humidity of 60% [62]. For the transcriptome sequencing, uniform rapeseed plants after 7-day seed germination were grown for 10 d under Cd-free condition, and then the seedlings were transferred to the solution containing low (0.50 μM) or high (50 μM) CdCl_2_ for three days until sampling, when the rapeseed plants start to show slight Cd toxicity symptoms under high Cd condition.

### Ionomic analysis

For the ionomic analysis, uniform rapeseed plants after 7-day seed germination were grown for 10 d under Cd-free condition, and then the seedlings were transferred to the solution containing 0.50 μM or 50 μM CdCl_2_ for five days until sampling, when the rapeseed plants start to show obvious Cd toxicity symptoms under high Cd condition. The shoot and root tissues of rapeseed plants were over-dried at 65 °C to constant weight. Subsequently, the samples that were ground to fine powder were transferred to a HNO_3_/HClO_4_ mixture (4:1, v/v) at 200 °C until the digestion was completed. The diluted supernatant was submitted to an inductively coupled plasma mass spectrometry (ICP-MS; NexIONTM 350X, PerkinElmer) to quantify the concentrations of mineral elements [30]. Each sample contained five independent biological replicates.

### RNA extraction

Total RNA was extracted from fresh rapeseed tissues using Invitrogen TRIzol® Reagent (Thermo Fisher Scientific, California, USA) according to the manufacturer’s instructions (Invitrogen), and genomic DNA was removed using DNase I (TaKara, Shiga, Japan). Then, RNA quality was determined by 2100 Bioanalyser (Agilent, Palo Alto, California, USA) and quantified using the NanoDrop 2000 (Thermo Fisher Scientific, Massachusetts, USA). Only high-quality RNA samples (OD_260_/OD_280_ = 1.8 ~ 2.2, OD_260_/OD_230_ ≥ 2.0, RIN ≥ 6.5, 28S:18S ≥ 1.0, > 2.0 μg) were used to construct sequencing library.

### Library preparation and sequencing

Transcriptome libraries were prepared following TruSeq™ RNA sample preparation Kit from Illumina (Illumina Inc., San Diego, California, USA) using 1.0 μg of total RNA. Shortly, mRNA was isolated according to poly-A selection method by oligo(dT) beads, and was then fragmented by fragmentation buffer. Secondly, double-stranded cDNA was synthesized using a SuperScript double-stranded cDNA synthesis kit (Invitrogen, CA) with random hexamer primers (Illumina, San Diego, California, USA). Then, the synthesized cDNA was subjected to end-repair, phosphorylation, and ‘A’ base addition according to Illumina’s library construction protocol. Libraries were size selected for cDNA target fragments of 200–300 bp on 2% Low Range Ultra Agarose followed by PCR amplified using Phusion DNA polymerase (NEB) for 15 PCR cycles. After quantification by TBS380, paired-end RNA-seq sequencing library was sequenced with the Illumina HiSeq xten/NovaSeq 6000 sequencer (2 × 150 bp read length).

### Read mapping

The raw paired end reads were trimmed and quality controlled by SeqPrep (https://github.com/jstjohn/SeqPrep) and Sickle (https://github.com/najoshi/sickle) with default parameters. Then, clean reads were separately aligned to reference genome with orientation mode using TopHat (http://tophat.cbcb.umd.edu/, version 2.0.0) [63]. The mapping criterion of bowtie was as follows: sequencing reads should be uniquely matched to the genome allowing up to 2 mismatches, without insertions or deletions. Then the region of gene was expanded following depths of sites and the operon was obtained. In addition, the whole genome was split into multiple 15 kb windows that share 5 k bp. New transcribed regions were defined as more than two consecutive windows without overlapped region of gene, where at least 2 reads mapped per window in the same orientation.

### Differential expression analysis and functional enrichment

To identify differentially expressed genes (DEGs) between different treatments, the expression level of each transcript was calculated according to the fragmets per kilobase of exon per million mapped reads (FRKM) method. The DEGs were defined as those with a *P* value and false discovery rate (FDR) that were less than 0.05 [64]. RSEM (http://deweylab.biostat.wisc.edu/rsem/) [65] was used to quantify gene expression abundances. R statistical package software EdgeR (Empirical analysis of Digital Gene Expression in R, (http://www.bioconductor.org/packages/2.12/bioc/html/edgeR.html) [66] was utilized for differential expression-n analysis. The Kaiser–Meyer–Olkin test was used for perform principal com-ponent analysis, to determine how many components were necessary to reduce the high-dimensionality transcriptome expression data using R statistical package (https://www.r-project.org/).

In addition, functional-enrichment analysis, including GO and KEGG, was performed to identify which DEGs were significantly enriched in GO terms and metabolic pathways at Bonferroni-corrected *P*-value ≤0.05 compared with the whole-transcriptome background. GO functional enrichment and KEGG pathway analysis were carried out by Goatools (https://github.com/tanghaibao/Goatools) and KOBAS (http://kobas.cbi.pku.edu.cn/home.do) [67].

### Gene co-expression network analysis

The interaction relationships of each gene pair were calculated based on the corresponding transcript abundance (FPKM value) using the online DeGNServer (http://plantgrn.noble.org/DeGNServer/) [68]. The parameter settings were as follows: (i) value-based co-expression network type, (ii) *Pearson* correlation estimation method, and (iii) association cutoff > 0.6. Gene co-expression networks were visualized by Cytoscape (http://www.cytoscape.org/) [69].

### Reverse transcription–quantitative polymerase chain reaction assays

Reverse transcription–quantitative polymerase chain reaction (RT-qPCR) assays were performed to validate the accuracy of transcriptome sequencing data. After removing genomic DNA from RNA samples with RNase-free DNase I, total RNA was used as RT templates for cDNA synthesis using the PrimeScript™ RT Reagent Kit Eraser (Perfect Real Time; TaKaRa, Shiga, Japan). The RT-qPCR assays were performed to detect relative gene expression using SYBR®*Premix Ex Taq*™ II (TliRNaseH Plus) (TaKaRa, Shiga, Japan) using a Bio-Rad C1000 touch Thermal Cycler of CFX96™ Real-time PCR detection System.

The RT-qPCR program was as follows: 95 °C for 3 min, 40 cycles of 95 °C for 10 s, and 60 °C for 30 s. The melting curve was plotted as follows to analyze the primer gene-specificity: 95 °C for 15 s, 60 °C for 1 min, and 60–95 °C for 15 s (+ 0.3 °C/cycle). The expression data of target genes were normalized using two public internal reference genes, *BnaEF1-α* and *BnaGDI1* [70], and the relative gene expression levels were calculated according to the 2^-ΔΔC^_*T*_ method [71]. Each sample contained three independent biological replicates.

### Statistical analysis

The Statistical Productions and Service Solutions 17.0 (SPSS, Chicago, IL, USA) was used to perform statistical tests. Student *t* test or one-way variance of analysis, followed by Tukey’s honestly significant difference multiple comparison tests, was used to determine the significance differences.

## Supplementary Information

**Additional file 1.**

## Data Availability

All the data and materials that are required to reproduce these findings can be shared by contacting the corresponding author, Dr. Ying-peng Hua (yingpenghua@zzu.edu.cn).

## Abbreviations

ABC: ATP-binding cassette
CAX: cation exchanger
Cd: cadmium
DEG: differentially expressed genes
HMA: heavy metal ATPase
IRT: iron-regulated transporter
MT: metallothionein
MTP: metal tolerance protein
NRAMP: natural resistance-associated macrophage proteins
PCR: plant cadmium resistance
PCS: phytochelatin synthetase;
YSL: yellow-stripe like
ZIP: ZRT/IRT-like protein
