# Supplementary material for: Integrated ionomic and transcriptomic dissection reveals the core transporter genes responsive to varying cadmium abundances in allotetraploid rapeseed
Source: BMC Plant Biol. 2021 Aug 13;21:372. doi: 10.1186/s12870-021-03136-w (PMC8362225; doi:10.1186/s12870-021-03136-w)
Supplement: Supplementary file 1 — Additional file 1. [file 12870_2021_3136_MOESM1_ESM.doc]

**Supplementary Table S1** Overview of the transcriptome sequencing data in this study

| Sample | | Raw reads | Raw bases | Clean reads | Clean bases | Mapped rate (%) | Q20  (%) | Q30  (%) | GC content (%) |
| --- | --- | --- | --- | --- | --- | --- | --- | --- | --- |
| Shoot | Mock | 50,838,673 | 7,676,639,573 | 50,344,435 | 7,470,487,371 | 89.90 | 97.98 | 93.97 | 48.72 |
|  | Low Cd | 46,831,341 | 7,071,532,541 | 46,046,565 | 6,783,414,487 | 90.87 | 97.89 | 93.78 | 47.68 |
|  | High Cd | 49,356,245 | 7,452,793,045 | 48,898,909 | 7,254,412,086 | 90.29 | 98.01 | 94.04 | 48.06 |
| Root | Mock | 46,370,145 | 7,001,891,945 | 45,917,199 | 6,817,138,695 | 90.87 | 97.72 | 93.31 | 47.32 |
|  | Low Cd | 47,697,903 | 7,202,383,403 | 47,231,710 | 7,000,992,582 | 90.65 | 97.96 | 93.94 | 47.34 |
|  | High Cd | 48,796,537 | 7,368,277,037 | 48,364,414 | 7,171,017,787 | 91.06 | 97.95 | 93.90 | 47.38 |

Mock mean the Cd-free condition.
